# Effects of plant-derived protein and rapeseed oil on growth performance and gut microbiomes in rainbow trout

**DOI:** 10.1186/s12866-023-02998-4

**Published:** 2023-09-13

**Authors:** Cunfang Zhang, Lingyong Hu, Jiahui Hao, Weijie Cai, Minxin Qin, Qiang Gao, Miaomiao Nie, Delin Qi, Rui Ma

**Affiliations:** 1 State Key Laboratory of Plateau Ecology and Agriculture, Qinghai University, Xining, 810001 China; 2 Northwest Institute of Plateau Biology, Chinese Academy of Sciences, Xining, 810001 China; 3 College of Eco-Environmental Engineering, Qinghai University, Xining, 810001 China

**Keywords:** Triploid rainbow trout, Diet, Microbe, Diversity, Network

## Abstract

**Background:**

Rainbow trout (*Oncorhynchus mykiss*) is becoming popular with the increased demand for fish protein. However, the limited resources and expense of fish meal and oil have become restrictive factors for the development of the rainbow trout related industry. To solve this problem, plant-derived proteins and vegetable oils have been developed as alternative resources. The present study focuses on evaluating the effects of two experimental diets, FMR (fish meal replaced with plant-derived protein) and FOR (fish oil replaced with rapeseed oil), through the alteration of the gut microbiota in triploid rainbow trout. The commercial diet was used in the control group (FOM).

**Results:**

Amplicon sequencing of the *16S* and *18S rRNA* genes was used to assess the changes in gut bacteria and fungi. Our analysis suggested that the α-diversity of both bacteria and fungi decreased significantly in the FMR and FOR groups, and β-diversity was distinct between FOM/FMR and FOM/FOR based on principal coordinate analysis (PCoA). The abundance of the Planctomycetota phylum increased significantly in the FMR group, while that of Firmicutes and Bacteroidetes decreased. We also found that the fungal phylum Ascomycota was significantly increased in the FMR and FOR groups. At the genus level, we found that the abundance of *Citrobacter* was the lowest and that of pathogenic *Schlesneria*, *Brevundimonas*, and *Mycoplasma* was highest in the FMR and FOR groups. Meanwhile, the pathogenic fungal genera *Verticillium* and *Aspergillus* were highest in the FMR and FOR groups. Furthermore, canonical correspondence analysis (CCA) and network analysis suggested that the relatively low-abundance genera, including the beneficial bacteria *Methylobacterium*, *Enterococcus*, *Clostridium, Exiguobacterium*, *Sphingomonas* and *Bacteroides* and the fungi *Papiliotrema*, *Preussia*, and *Stachybotrys,* were positively correlated with plant protein or rapeseed oil. There were more modules that had the above beneficial genera as the hub nodes in the FMR and FOR groups.

**Conclusions:**

Our study suggested that the FMR and FOR diets could affect the gut microbiome in rainbow trout, which might offset the effects of the dominant and pathogenic microbial genera. This could be the underlying mechanism of explaining why no significant difference was observed in body weight between the different groups.

**Supplementary Information:**

The online version contains supplementary material available at 10.1186/s12866-023-02998-4.

## Background

Fish consumption has recently shown high growth trend [1]. Triploid rainbow trout (*Oncorhynchus mykiss*) is one of the most successful commercial fish, providing more than 30,000 tons of products per year. Furthermore, an approximately 45.8% protein content is required in the optimal diet of rainbow trout [2]. The protein in commercial feed is mainly provided by fish, and the lipids are mainly provided by fish oil, which is expensive and limited. Therefore, some studies have explored the replacement of fish protein with plant protein sources, insect protein, yeast protein, and so on [3–5]. Among these, plant proteins are the main alternative source for fish meal. It has been proven that replacement with full plant-derived protein in the fish diet has no effect on fish growth performance [3, 6]. This provides an alternative avenue for using plant protein instead of fish meal for rainbow trout feeding.

It is well known that the gut microbiota is most sensitive to diet and plays an important role in metabolizing nutrients and maintaining the health of fish [7, 8]. Studies have confirmed that the gut microbes of herbivorous and carnivorous animals, including fishes, are largely different [8–11]. At the phylum level, the Bacteroidetes are dominant in carnivorous animals, whereas Firmicutes are the primary microbes in herbivores. However, the effects of dietary adjustments on fish growth performance and gut microbes, there were some similarities, some of which are related to dietary alternatives. For example, in European Sea Bass (*Dicentrarchus labrax*), replacement of 15% protein of a vegetal formulation with insect or yeast proteins led to significantly higher fish growth performance than with the full vegetal formulation, with a feed conversion ratio similar to that of a commercial diet. The gut microbial community showed predominance of the phyla Proteobacteria, Firmicutes, Actinobacteria, and Bacteroidetes. The partial replacement of the protein source in the diet was not associated with differences in fish gut microbial richness [12]. In grass carp (*Ctenopharyngodon idellus*), both the water and alcohol extracts of faba bean can increase body weight and the diversity of intestinal microbes, but protein extract and residue diets had no effect on body weight gain and caused intestinal floral disorder, respectively [13]. A study in Nile tilapia (*Oreochromis mossambicus*) showed that Planctomyetes was the main phylum in juvenile fish fed plant-based foods, which had a negative effect on tilapia survival and gut development [14]. In red drum (*Sciaenops Ocellatus*), fish fed a high-plant-protein diet showed markedly increased production performance and survival rates, and the main phyla were Firmicutes, Bacteroidetes, Fusobacteria and Actinobacteria; among them, Fusobacteria includes some pathogenic bacteria [15]. In juvenile turbot (*Scophthalmus maximus*), dietary fat levels are very sensitive to the intestinal microbiota and intestinal health, and a medium lipid diet (12%) was more conducive than other diets to fish gut health and microbiota stability [13].

These results suggested that significant changes in the dietary composition of either protein or fat could affect fish growth performance and gut microbes. The influence has both advantages and disadvantages. Triploid rainbow trout is a typical carnivorous fish. Changes in the diet from carnivory to phytophagy will have an effect on intestinal microorganisms. Pérez-Perez-Pascual et al. [12] reported that the gut bacterial richness and growth performance of rainbow trout can improve significantly when a vegetal protein diet is partially replaced with yeast or insect proteins without affecting health. In the present study, to further reduce feed costs and maintain rainbow trout growth performance, we replaced all fish meal with plant protein and all fish oil with rapeseed oil in diets and then evaluated effects on the growth performance and intestinal microbiota of rainbow trout. Our results provide an experimental basis and useful guidance for rainbow trout farming.

## Results

### Diet has no effect on the growth performance of rainbow trout

After 84 days of feeding with the three different diets (FOM, a commercial fishmeal-rich diet; FMR, a diet in which fish meal was replaced with plant protein; FOR, a diet in which fish oil was replaced with rapeseed oil (Supplementary Table 1)), the average body weight and body length of rainbow trout were 793.33 ± 73.18 g and 35.83 ± 1.25 cm, 831.66 ± 124.82 g and 34.66 ± 2.62 cm, 758.33 ± 46.7 g and 33.83 ± 1.25 cm in the fish fed with the control diet (FOM), FMR, and FOR, respectively (Supplementary Table 2). Comparisons of the body weight revealed that the average mean of the FMR group was highest, followed by FOM, and FOR; however, the differences were not significant. There were slight differences in body length among the three diet groups.

### Summary of high-throughput sequencing

**Fig. 1:**
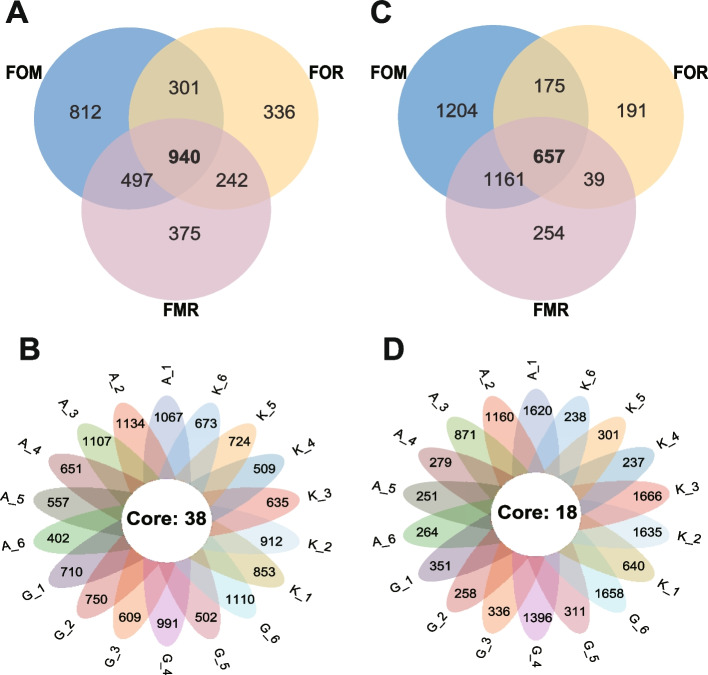
Venn and flower diagram analysis of shared OTUs. **AC** Venn diagram shows the number of OTUs that were shared by the fish fed the FOM, FMR and FOR diets. **B** **D** Flower diagram shows the number of cores OTUs that were shared by 18 samples from the FOM (from A-1 to A-6), FMR (from G-1 to G-6) and FOR (from K-1 to K-6)

To assess the effects of diet changes on bacteria and fungi, we performed *16S rRNA* V3-V4 region and ITS1 region sequencing and analyzed the microbial gut content of rainbow trout fed three diets. An average of 46,086 and 64,407 reads and an average length of 403 bp and 255 bp for the amplified *16S rRNA* and *ITS* genes were obtained from each sample. After quality filtering and chimera removal, we obtained an average of 33,606 and 64,326 good-quality reads, respectively (Supplementary Table 3). Meanwhile, the sequences of the *16S rRNA* gene annotated as chloroplastics were removed, and diversity analysis was performed according to the sample depth with the fewest reads (8476) and 53,110 reads in fungi. In total, all 3503 Operational taxonomic units (OTUs) of the *16S rRNA* gene were obtained from 18 samples and sorted into 48 phyla, 142 classes, 329 orders, 573 families and 1180 genera. Meanwhile, all OTUs of the ITS gene were obtained from the same samples and were sorted into 15 phyla, 50 classes, 125 orders, 287 families and 569 genera. A Venn diagram was used to identify the common gut microbes in the fish treated with different diets. The results suggested that 940 bacterial OTUs were shared by different diet groups, and the 38 core OTUs were shared by 18 samples (Fig. 1A and B). Interestingly, there were 657 common fungal OTUs in the fish fed different diets and 18 core OTUs in 18 samples (Fig. 1C and D).

### Diet affects the α-diversity and β-diversity of rainbow trout gut bacteria and fungi

**Fig. 2:**
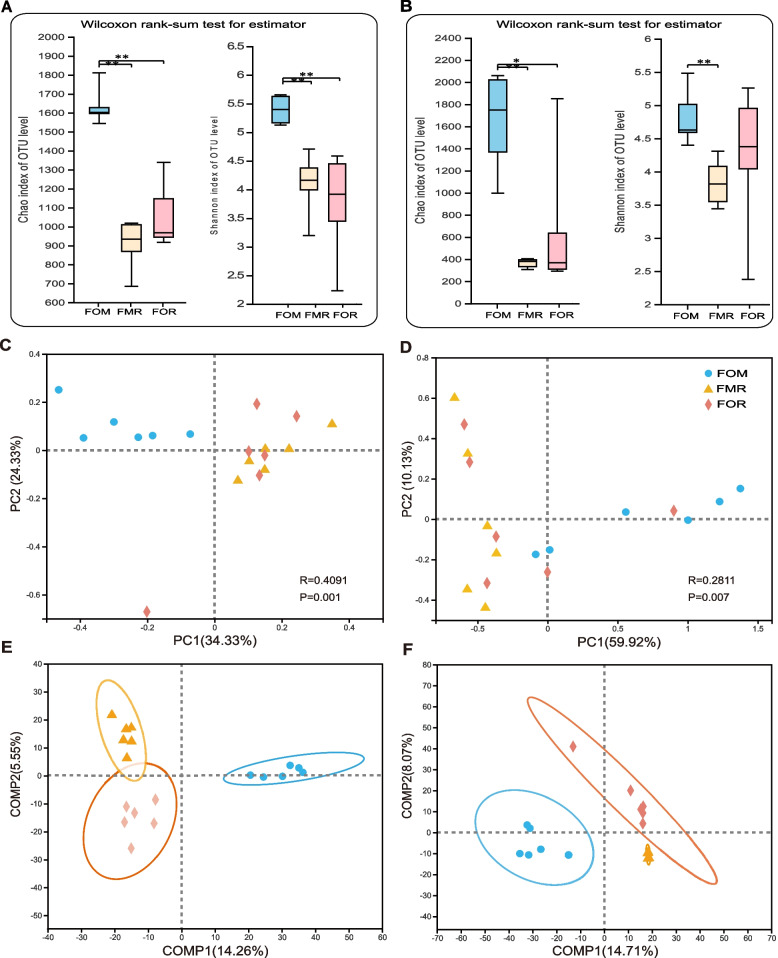
Bacterial and fungal α-diversity and β-diversity of the gut of rainbow trout submitted to three different diets (*n* = 6 fish/diet). **A** Bacterial α-diversity metrics and **B** fungal α-diversity metrics. **C** **D** Principal coordinate analysis (PCoA) analysis based ons Bray–Curtis distance of the bacterial and fungal community of different three diets. **E** **F** PLS-DA analysis of the bacterial and fungal communities in the three different diets. FOM was the control diet, a commercial-like diet containing fishmeal and fish oil, and in the FMR group, fishmeal was replaced completely with plant protein. For the FOR group, fish oil was replaced completely with rapeseed oil. Significance: *, *p* value < 0.05; **, *p* value < 0.01

Compared with those of the fish in the FOM (Fig. 2A and B), the intestinal bacteria and fungi of fish in the FMR and FOR had decreased Chao indices (FOM vs. FMR and FOM vs. FOR, *p* values = 0.005 and 0.005; *p* values = 0.005 and 0.02, respectively), the Shannon indices of bacteria also decreased significantly (FOM vs. FMR and FOR, *p* values = 0.005 and 0.005; *p* values = 0.02 and 0.230, respectively), and those of fungi decreased significantly in FOM vs. FMR (*p* values = 0.005). There richness and species diversity of both bacteria and fungi were significantly lower in fish fed the replacement diets FMR and FOR. Principal coordinate analysis (PCoA) was employed to evaluate the differences in the gut bacterial and fungal community structures among the three diets based on Bray–Curtis distance metrics (Fig. 2). As shown in Fig. 2C and D, the gut bacterial and fungal communities in the fish fed the three diets all showed significant differences (Adonis R^2^ = 0.409, *p* = 0.001; Adonis R2 = 0.281, *p* = 0.007, respectively). However, except for the gut bacterial community for the FOM diet, which formed a single cluster, there was crossover between samples of the gut bacterial community for the FMR and FOR diets and the gut fungal community for the three diets. Therefore, we performed PLS-DA analysis to further distinguish between these three groups, and the results suggested that the gut bacterial and fungal communities of the three diet groups could all be clustered individually by dietary group (Fig. 2E and F).

### Diet affects the community composition of rainbow trout gut bacteria and fungi

**Fig. 3:**
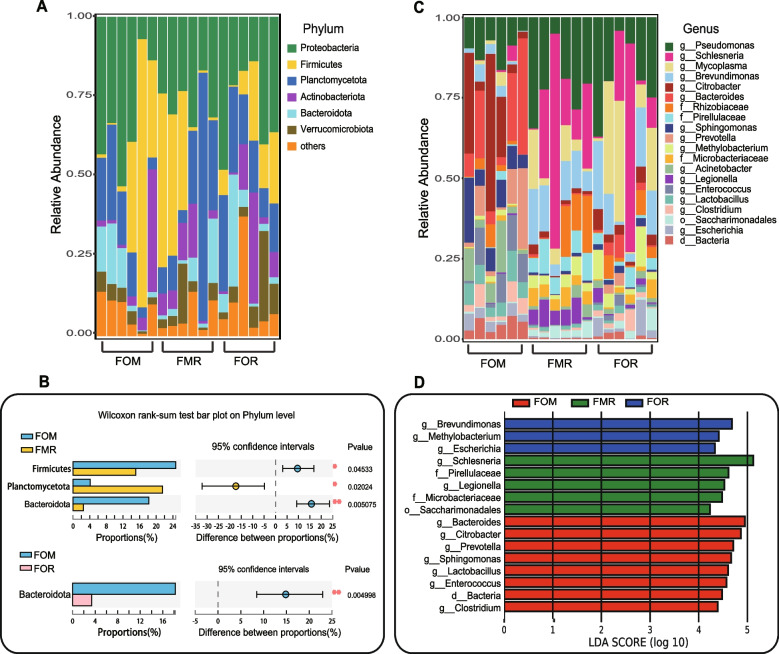
Relative abundance (%) of the overall most prevalent bacterial phyla and genera of the gut of rainbow trout fed different diets and Wilcoxon rank-sum test for differences between groups. **A** Bar charts show the abundance of the top 6 phyla. **B** Significant differences between the FOM and the replacement diets (FMR and FOR) at the phyla, respectively, marked with an asterisk (*, *p* < 0.05, **, *p* < 0.01). **C** Bar charts show the abundance of the top 20 genera. **D** LEfSe analysis at the genus levels. Note: g_Escherichia is the g_Escherichia-Shigella

A total of 48 bacterial phyla were detected, among which Proteobacteria, Firmicutes, Planctomycetota, Actinobacteriota and Bacteroidota were the top 5 abundant phyla (Fig. 3A). We found that fish fed the FMR diet showed a significant increase in the relative abundance of the phylum Planctomycetota (FMR: 21.51% vs. FOM: 4.41%, *P* = 0.020) and a significant decrease in Firmicutes (FMR: 15.07% vs. FOM: 24.70%, *P* = 0.045) and Bacteroidetes (FMR: 2.49% vs. FOM: 18.22%, *P* = 0.005) compared to fish fed FOM (Fig. 3B and Supplementary Fig. 1). For the group fed rapeseed oil (FOR), the relative abundance of only the phylum Bacteroidetes (FMR: 3.38% vs. FOM: 18.22%,* P* = 0.005) decreased significantly compared to that in the fish fed the control diet (FOM). At the genus level, *Pseudomonas* was the dominant genus in all diet groups, and *Citrobacter* had the highest abundance in the FOM group, but its abundance was very low in both the FMR and FOR groups. *Schlesneria*, *Brevundimonas* and *Mycoplasma* were the dominant genera in the FMR and FOR fish groups. In particular, the abundance of *Schlesneria* was the highest but was lower and negligible in the FOM group (Fig. 3C). Further LEfSe analysis (LDA score > 4) showed that the three groups had significantly different bacterial genera; there were 8, 3, and 5 different bacterial species in the FOM, FOR, and FMR groups, respectively. Only *Schlesneria* had an LDA value greater than 5 in the FMR group (Fig. 3D).

**Fig. 4:**
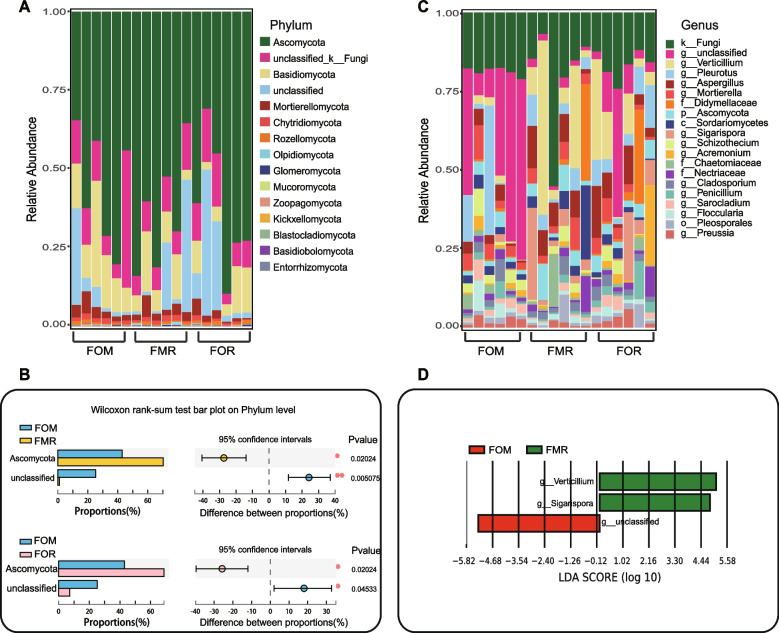
Relative abundance (%) of the overall most prevalent fungal phyla and genera of the gut of rainbow trout fed with different diets and Wilcoxon rank-sum test for differences between groups. **A** Bar charts show the abundance of all phyla. **B** Significant differences between FOM and the replacement diets (FMR and FOR) at the phyla, respectively, marked with an asterisk (*, *p* < 0.05, **, *p* < 0.01). **C** Bar charts shown the abundance of the top 20 genera. **D** LEfSe analysis at the genus levels.s

A total of 15 different fungal phyla were identified in the three diet groups, among which Ascomycota, Basidiomycota, Mortierellomycota and Chytridiomycota were dominant (Fig. 4A and Fig. S1). Compared with that in the control fish (FOM), the phylum Ascomycota was significantly increased in fish fed the plant protein diet (FMR, 70.27%, *P* = 0.020) and the rapeseed oil diet (FOR, 68.84%, *P* = 0.020) (Fig. 4B and Fig. S1). At the genus level, k_Fungi, an unknown genus of fungi, was the dominant genus in all three groups. *g_unclassified* was the dominant genus in the FOM and FOR groups (Fig. 4C), and *Verticillium* and *Aspergillus* were the dominant genera in the FMR and FOR groups, both of which had plant-derived components in their diets (Fig. 4C). The LEfSe analysis (LDA score > 4) showed that there were 3 significantly different genera among all groups, *among which Verticillium* and *Sigarispora* were the different genera in the FMR group, *and g_unclassified* was the different genus in the FMR group (Fig. 4D).

### Positive correlations between dietary components and the fish gut microbiota

To evaluate the effects of dietary components on the gut microbiota of rainbow trout, we performed canonical correspondence analysis (CCA) with fish meal, plant proteins, fish oil and rapeseed oil as explanatory variables and 20 dominant bacterial and fungal community members as response variables (Fig. 6A). The CCA results showed correlations between genera and the four diet components. In the bacterial community, *Escherichia-Shigella* and *Clostridium* were obviously positively related to fish meal; *g_norank_f_Rhizobiales_Incertae_Sedis* and *Legionella* were obviously positively related to plant proteins; and *Mycoplasma* and *Methylobacterium* were obviously positively related to rapeseed oil. In the fungal community, *Acremonium*, *Penicillium* and *o_Pleosporales* were obviously positively related to rapeseed oil; *Cladosporium*, *Sarocladium* and *Preussia* to fish meal; *g_unclassified_f*_*Chaetomiaceae* and *g_unclassified_c_Sordariomycetes* to plant protein; and *g_unclassified_k_Fungi* to fish oil.

### Effect of different diets on gut bacterial and fungal networks

The gut bacterial and fungal networks were constructed with clusters as modules, and the average clustering coefficient was greater than 0.5 for each diet (Supplementary Table 4). As shown in Fig. 6A and B, the network of gut bacterial and fungal communities for each diet showed distinct co-occurrence patterns. The fish fed the FOM diet had the fewest (three) modules in both the fungal and bacterial networks, while the FMR and FOR groups had at least six modules (Fig. 6A, B, Supplementary Table 4). In addition, fish fed the control diet (FOM) had a more complex network than those fed the rapeseed oil diet (FOR), and the network of FOR was more complex than that of the plant protein diet (FMR), as FOM and FOR possessed more links (Fig. 6A, B, Supplementary Table 4). Meanwhile, positive associations were greater than negative associations for all three diet groups in both the fungal and bacterial networks, which is consistent with many other results. According to the ranking of degrees (Supplementary Table 4), the central nodes of different modules were found in each network and are listed in Table 1. In the bacterial network, *Treponema*, *Pontibacter*, *Acinetobacter*, etc., were the hub nodes of the three modules of fish fed the FOM diet, which mainly belong to Proteobacteria and Bacteroidetes. *Thermomonas*, *Enterococcus*, *Desulfitibacter*, *Exiguobacterium*, *Denitratisoma*, *Sphingomonas*, *Rhodanobacter*, *Bacteroides*, etc., were the hub nodes of modules 1–6 of the FMR group; Enterococcus belongs to Firmicutes and is included among the main phyla compared with those in the FOM group; *Olsenella*, *Leptotrichia*, *Bryobacter*, *Bdellovibrio*, *Exiguobacterium*, *Romboutsia*, *Pigmentiphaga*, *Clostridium*, *Exiguobacterium*, *Romboutsia*, *Parvimonas*, etc., were the hub nodes of modules 1–7 of the FOR group, which mainly belong to the phyla Actinobacteriota, Fusobacteriota Acidobacteriota, Bdellovibrionota, Firmicutes, and Proteobacteria.

**Table 1:** The Modules, degree and hub nodes of three diets both in fungal and bacterial networks

	**Bacteria**			**Fungi**		
	**Modules**	**Degree**	**Hub nodes**	**Modules**	**Degree**	**Hub nodes**
**FOM**	module1	54	Treponema	module1	30	unclassified_f__ Didymellaceae
	module2	47	Pontibacter, Gemmatimonas	module2	35	Chaetomium,Filobasidium
	modele3	39	Acinetobacter, Neisseria, Massilia and SM1A02	modele3	27	Pichia
**FMR**	module1	15	Thermomonas	module1	13	unclassified_o__ Branch06
	module2	28	Enterococcus, Comamonas	module2	18	Aspergillus, Sarocladium
	modele3	14	Denitratisoma, Allorhizobium-Neorhizobium- Pararhizobium-Rhizobium	modele3	17	Papiliotrema
	module4	18	Sphingomonas, Psychrobacter, Exiguobacterium	module4	11	unclassified_p__ Ascomycota, Bipolaris
	module5	9	Rhodanobacter, Desulfitibacter	module5	13	Preussia, Pichia
	modele6	25	Bacteroides	modele6	21	Stachybotrys
**FOR**	module1	9	Olsenella, Aeromonas	module1	15	Zopfiella, Metarhizium
	module2	22	Leptotrichia, Azospira	module2	30	unclassified_c__GS13
	modele3	19	Bryobacter	modele3	16	unclassified_f__ Ceratobasidiaceae
	module4	22	Bdellovibrio, Clostridium_sensu_stricto_13, norank_f__Gemmatimonadaceae	module4	31	Plectosphaerella
	module5	19	Exiguobacterium	module5	19	Alfaria
	modele6	11	Romboutsia norank_f__Rhizobiales_ Incertae_Sedis	module6	6	Hyphopichia, Fusicolla, Bipolaris, Entrophospora, Simplicillium
	modele7	1	Pigmentiphaga, Parvimonas			

In the fungal network, *unclassified_f_Didymellaceae, Chaetomium*, *Pichia*, etc., were the hub nodes of FOM fish, which mainly belong to the phylum of Ascomycota. *Unclassified_o_Branch06*, *Aspergillus*, *Papiliotrema*, *unclassified_p_Ascomycota*, *Preussia*, *Stachybotrys*, etc., were the hub nodes of the FMR group, which mainly belong to the phyla Ascomycota and Basidiomycota. *Zopfiella*, *unclassified_c_GS13*, *unclassified_f__Ceratobasidiaceae*, *Plectosphaerella*, *Alfaria*, *Hyphopichia*, etc., were the hub nodes of the FOR group, which mainly belongs to the phyla Ascomycota, Chytridiomycota, and Basidiomycota.

## Discussion

### Plant-derived protein and oil diets have no effect on the growth performance of rainbow trout

With the development of fisheries, it is necessary to replace fish oil and fish meal with plant-derived protein and oil or other sources to reduce feeding costs. An increasing number of studies have focused on alternative feed and its effects on fish growth. Plant-derived proteins have been employed to replace fish meal for many fish species. Soybean meal is the best plant substitute for protein [13, 16]. However, the addition of soybean meal has a maximum limit because of the adverse effects of its anti-nutritional factors in carnivorous fish [17]. Studies have suggested that feeding fish plant-source protein improve [4, 15] or has no effect [3, 6, 13] on their growth compared to that of fish fed a control diet. Compared with the commercial feed group (FOM), there was no significant difference in body weight in the FMR group or the FOR group after feeding for three months (Supplementary Table 2). However, some studies have shown that the daily feed utilization rate is lower in fish fed a full vegetal diet than in those fed a commercial diet, which affects the growth performance of rainbow trout [12]. Moreover, in California yellowtail (*Seriola dorsalis*), compared with that under a commercial diet, the weight gain was equal in fish fed with and without fish meal, and fish oil was replaced with an algal oil diet [18]. These inconsistent results are likely due to the differences in the fish oil, algal oil and rapeseed oil, which offer the essential fatty acids and may regulate the absorption of vegetable protein.

### Plant-derived protein and oil diets decreased the diversity of gut bacteria and fungi in rainbow trout

Although these results provide support for using vegetable protein or canola oil to completely replace fish meal and fish oil in the fish diet, they are far from sufficient, and many other factors need to be considered. Recent studies have suggested that the gut microbiome is essential for nutrient absorption and the health of fish [19–21]. In this study, we assessed the effects of FMR and FOR diets on the intestinal microbiota of triploid rainbow trout. In terms of α-diversity, the two experimental groups (FMR and FOR) showed significantly reduced values compared with those of the FOM group (Fig. 2A), as well as for fungi (Fig. 2B). The β-diversity showed that the PC values were relatively low for bacteria and fungi (Fig. 2C and D), which might be affected by environmental and host factors [8, 9, 11]. There were significant differences among the three groups; in particular, the difference between the two experimental groups (FMR and FOR) and the control group (FOM) was obvious in terms of the intestinal microbes of rainbow trout. This might be caused by the complete replacement of fish meal and fish oil with plant-derived protein and oil.

### Diets have significant effect on the dominant gut flora in rainbow trout

In several studies, fish meal and/or fish oil in commercial diets was completely replaced by plant-derived protein and oil, and the results were largely similar to those of our study on bacteria. Little research has been conducted on the effects of diet on gut fungi in fish. Naya-Català et al. [22] reported that the relative abundance of fungi increased slightly in fish fed a plant-based diet compared to fish fed a commercial diet. In general, animal gut microbial community composition shows large differences between individuals or within groups, as was the case in this study (Fig. 3A and C); however, the Adonis test showed that R > 0 and *p* < 0.05 (Fig. 2C and D), which indicates that the difference between groups was significantly larger than the difference within groups, therefore we focused on differences between groups in this study. Regarding microbial community composition, the phyla Proteobacteria, Firmicutes, Bacteroidetes, and Fusobacteria are the predominant gut bacteria in most fish species [19]. In *Oncorhynchus mykiss*, Actinobacteria is also a dominant bacterial phylum [12, 23–26]. Except for Fusobacteria, these phyla and Planctomycetota were the dominant phyla (Fig. 3A), and Planctomycetota was also dominant gut bacterial phylum of three species of cold-water fish in the Upper Yangtze River [27]. We speculated that this difference might be related to the living water environment because fish gut bacteria mainly come from this environment, and the enrichment of bacteria in species with different diets is conducive to food digestion. Therefore, fish with similar diets in similar environments have similar gut microbes. It is worth noting that the abundance of Planctomycetota increased significantly, while that of Firmicutes and Bacteroidetes decreased greatly in the FMR group compared to the FOM group (Fig. 3C and Fig. S1). Similar results were found in studies of three species of cold-water fish, Planctomycetota was higher and Firmicutes was lower in the herbivore *Schizothorax wangchiachii* than in the omnivorous *Schizothorax kozlovi* and carnivore *Percocypris pingi* [27]. Therefore, these two bacterial *phyla* might be related to the digestion of plant-derived diets. The fungal *phyla* Ascomycota, Basidiomycota, Mortierellomycota and Chytridiomycota were the dominant phyla in our study (Fig. 3B and Fig. S1), and Ascomycota and Basidiomycota are pathogens in a variety of marine animal hosts [28]. Ascomycota abundance was significantly increased in the FMR and FOR groups (Fig. 3D and Fig. S1), suggesting that diets including plant-derived components were not effective for carnivorous rainbow trout. Indeed, there were corresponding results at the genus level, *Pseudomonas, Brevundimonas, Schlesneria* and *Mycoplasma* were significantly more abundant in the experimental FMR and FOR groups than in a low-starch or commercial diet group, and they were reported as the main pathogenic microorganisms of fish [29–31]. However, *Citrobacter*, belonging to the Proteobacteria phylum, was significantly less abundant in the experimental FMR and FOR groups (Fig. 4A and B), which could increase the energy harvest in fish [31]. Among fungi, *Verticillium* and *Aspergillus* belong to the Ascomycota phylum, and *Aspergillus*, can cause fish feed and fish product deterioration [32]. Therefore, the addition or substitution of plant-derived components for fish meal may lead to an increase in the abundance of pathogenic bacteria and fungi, which could be detrimental to carnivorous fish.

### Correlation between main dietary components and main gut microbies in rainbow trout

**Fig. 5:**
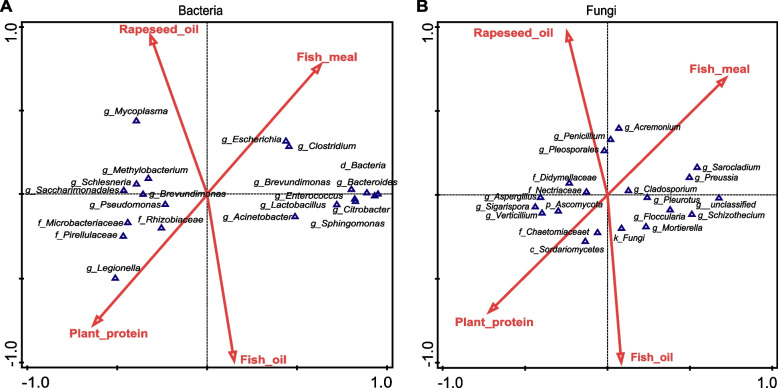
Canonical correspondence analysis (CCA) for the gut bacterial **A** and fungal **B** composition comparisons between rainbow trout fed the three different diets (FOM, FMR and FOR) for three months (*n* = 6 fish/diet); FOM: commercial diet including fishmeal and fish oil, FMR: fish meal replaced with plant proteins, FOR: fish oil replaced with rapeseed oil. Fish meal, plant proteins, fish oil and rapeseed oil were the four different components of the three diets and were regarded as factors to analyze correspondence with the 20 dominant genera from gut bacteria and fungi. Note: g_Escherichia is the g_Escherichia-Shigella

To further analyze the correlation between diet components and microbial community composition, we performed a CCA between diet components and bacterial genera. In the present work, *Escherichia-Shigella* and *Clostridium* were positively related to fish meal (Fig. 5A), and according to the abundance analysis, the abundance of *Escherichia* and *Clostridium* in the FOM and FOR diets containing fish meal was also significantly higher than that in the FMR diet without fish meal (Fig. 4A and B). In contrast, *Escherichia-Shigella* showed little change, and *Clostridium was* less abundant in fish fed a commercial diet compared to those fed a full vegetal diet [33]. However, there were consistent results in another two studies, in which the *Escherichia-Shigella* and *Clostridium* genera had a significantly higher abundance in fish fed a commercial diet than in fish fed a 40% soybean diet [23, 34]. Members of the *Escherichia* and *Clostridium* genera are common effective microorganisms in aquaculture [35–39]. In addition, *Mycoplasma* was positively related to rapeseed oil, and *Methylobacterium was* positively related to rapeseed oil and plant protein (Fig. 5A). *Mycoplasmas* are present more frequently in carnivorous fish than in other types of fish [40]. Another *Methylobacterium* genus was also obviously more abundant among the gut bacteria of European sea bass fed a diet mixed with plant-derived protein and rapeseed oil [4, 41]. These results indicated that *Methylobacterium* was highly abundant in fish fed plant-derived diets, which might be related to its ability to degrade carbohydrates and short-chain fatty acids. Similarly, the fungal genera *Acremonium, Penicillium* and *Pleosporales* were positively correlated with rapeseed oil (Fig. 5B), which are pathogenic fungi, and the *Acremonium* genus causes diseases of fish and shellfish [42]. The fungal genera were positively associated with plant proteins (Fig. 5A), for example, *g_unclassified*_*f_Chaetomiaceae* has a strong cellulose decomposition ability and bacteriostatic activity [43]. For both bacteria and fungi in the FMR and FOR groups, the dominant members are mostly pathogenic. Why does this phenomenon have no significant impact on the health and growth of fish?

**Fig. 6:**
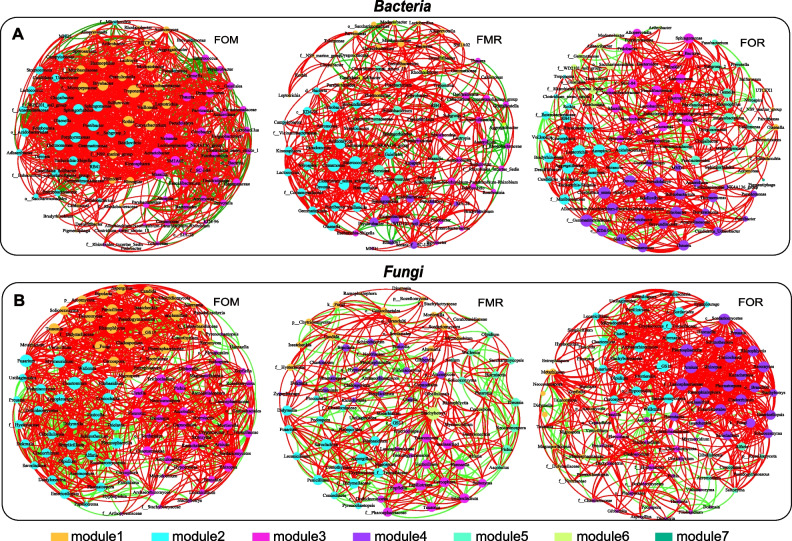
Co-occurrence networks of the top 100 genera in gut bacteria **A** and fungi **B** among different diet groups. Nodes represent genera, and their sizes indicate different relative abundances. Links between the nodes represent a significant and strong correlation between 2 genera (Spearman’s correlation greater than 0.7 or lower than − 0.7). Line color reflects direction (green: negative; red: positive). FOM: commercial diet including fish meal and fish oil, FMR: fish meal replaced with plant proteins, FOR: fish oil replaced with rapeseed oil

Gut microbes participate in host physiology in the form of a network. In addition to analyzing the changes in dominant phyla and genera for different diet groups, network relationship analysis among microorganisms was also performed. As explained in the majority of mammalian studies, the complexity of the gut microbiota network is positively correlated with the stability of the host gut microbiota community structure and feed utilization [44]. The network of gut bacteria is more complex in omnivorous and carnivorous fish than in herbivorous fish [27]. In the present work, the correlations of the gut microbiota were mainly positive with the three different diets, and there were fewer modules and less complexity in the networks of the gut microbiota of in the FOM group than in the other two groups (Fig. 6). These results indicated that the stability of the intestinal flora was disrupted and the utilization rate of feed was reduced in rainbow trout when the conventional commercial diet was replaced with plant-derived protein and rapeseed oil. However, the growth of fish in the FMR and FOR groups was not affected. This might be explained by the fact that there were more network modules among the gut microbiota in these two groups*.* As shown in Table 1, more genera and phyla of bacteria and fungi were detected in fish fed the plant protein diet (FMR) and the rapeseed oil diet (FOR) than in fish fed the control diet (FOM, a commercial-like diet containing fishmeal and fish oil). Therefore, we speculated that carnivorous rainbow trout need more microbial assistance in digesting plant-based protein and fat. Moreover, the modules with *Enterococcus* [45], *Clostridium* [36, 37], *Exiguobacterium* [46, 47], *Sphingomonas* [48], and *Bacteroides* [49] as hub nodes might be beneficial for the utilization of the plant diets and the maintenance of fish intestinal microfloral stability in the FMR group, as some studies have confirmed that species from these bacterial genera are beneficial to fish. For example, the *Enterococcus* genus could enhance fish growth, modulate the gut microbiota to suppress inflammatory responses, and boost the immune system [45, 50]. In fungi, the hub nodes formed by the *Papiliotrema, Preussia,* and *Stachybotrys* genera played an important role in metabolism, fermentation and anti-inflammation [51]. Therefore, it is reasonable to believe that the network modules formed by these low-abundance microbes offset the effects of plant-derived protein and rapeseed oil substitution in the FMR and FOR diets.

## Conclusion

The present study revealed that replacing the fishmeal and fish-oil in a commercial diet with plant-derived protein and that rapeseed oil had no significant effect on growth performance. It had some influence on intestinal microorganisms, and several pathogenic bacteria and fungi were dominant in the FMR and FOR groups; however, there were also more modules and multiple nondominant beneficial bacteria and fungi forming network modules. This result suggested that it is possible to use the plant-derived protein and rapeseed oil to completely replace the fishmeal and fish oil in the commercial diet of triploid rainbow trout.

## Methods

### Fish experiment

Three kinds of diets were produced by Tongwei Group Co., Ltd., China. (Supplementary Tables 1 and 2). The FOM fish were fed the control diet, a commercial-like diet containing fish meal and fish oil. In the FMR group, fish meal was replaced completely with plant protein. In the FOR group, fish oil was replaced completely with rapeseed oil. Rainbow trout from Longyangxia Reservoir of Qinghai Province in China were used in the present study. Feeding experiments were also carried out at Longyangxia Reservoir, and the local altitude was 2600 m. A total of 1200 fish (initial average weight of 208.34 ± 0.22 g) were randomly divided into 3 groups according to diet with 4 cages in each group and 100 fish per cage (the cage size was 3 × 3 × 6 m^3^). Fish were hand-fed to apparent satiation twice daily at approximately 8:30 a.m. and 16:30 p.m., the trial lasted 84 days from September to December, during which the average water temperature was approximately 10 ℃.

### Growth index measurement and sampling of intestinal contents

The fish were starved overnight after the 84-day trial, and the intestinal contents of the rainbow trout were collected for gut microbiome analysis. First, all fish were weighed and measured, the blood and viscera weights were subtracted to obtain the final body weight, and the body length was measured from the front of the jaw to the base of the caudal fin. Then six fish were selected randomly from each diet group and euthanized by an overdose of 300 mg/L tricaine methane sulfonate (MS-222, Sigma). The fish were disintegrated, the whole intestine was separated and washed with sterile PBS 3 times, and all the intestinal contents were extruded into 15 mL cryopreservation tubes, stored in liquid nitrogen, and then transferred to − 80 °C for storage. A total of 18 intestinal content samples were collected.

### DNA extraction and amplicon sequencing

Total DNA was extracted from 18 samples using the Stool DNA Kit (Omega, M4015-01), after which DNA integrity was tested by 0.8% agarose gel electrophoresis, and the concentration and purity were measured by a NanoDrop 2000 instrument (Thermo Scientific Ltd.). Samples with DNA concentrations > 8 ng/μL and absorbance values at 260: 280 > 1.8 were used for further analysis. The primers 341F: 5′-CCTACGGGNGGCWGCAG-3′ and 805R: 5′-GACTACHVGGGTATCTAATCC-3′ were employed to amplify the 16S rRNA V3-V4 region (460 bp) of bacteria, and the primers ITS1F: 5′-CTTGGTCATTTAGAGGAAGTAA-3′ and ITS2R: 5′-GCTGCGTTCTTCATCGATGC-3′ were used to target the ITS1 region (350–570 bp) of fungi. The PCR system consisted of 10–20 ng of genomic DNA, 15 µL of 2 × Taq Master Mix (Vazyme, P111-03), 1 µL of each primer (10 µM) and ddH_2_O to a total volume of 30 μL. PCR amplification of the two regions was performed as follows: initial denaturation at 95 °C for 3 min, followed by 30 cycles of denaturing at 95 °C for 30 s, annealing at 55 °C for 30 s and extension at 72 °C for 45 s, a single extension at 72 °C for 10 min, and end at 4 °C. For the purification and recovery of PCR products, fragments > 400 bp with 0.6 magnetic beads (Agencourt AMPure XP, Transgen) were selected for treatment in bacteria, and fragments < 400 bp with 0.8 magnetic beads were selected for treatment in fungi. The purified amplicons were quantified accurately using a Qubit 3.0 DNA detection kit (Life, Q10212). Finally, purified amplicons were pooled in equimolar amounts and paired-end sequenced on the Illumina MiSeq PE300 platform (Illumina, San Diego, USA) according to the standard protocols by Majorbio Bio-Pharm Technology Co. Ltd. (Shanghai, China).

### Sequence data processing and statistics

The raw reads from *16S rRNA* gene and *ITS* gene (PRJNA1003964) sequencing were demultiplexed, quality-filtered by fastp version 0.20.0 and merged by FLASH version 1.2.7 with the following criteria: (i) the 300 bp reads were truncated at any site receiving an average quality score of < 20 over a 50 bp sliding window, and the truncated reads shorter than 50 bp were discarded. Reads containing ambiguous characters were also discarded. (ii) Only overlapping sequences longer than 10 bp were assembled according to their overlapped sequence. The maximum mismatch ratio of the overlap region was 0.2. Reads that could not be assembled were discarded. (iii) Samples were distinguished according to the barcode and primers, and the sequence direction was adjusted, with exact barcode matching and 2 nucleotide mismatches in primer matching. Operational taxonomic units (OTUs) were clustered with a 97% similarity cutoff using UPARSE version 7.1, and chimeric sequences were identified and removed. The taxonomy of each representative OTU sequence was assigned by RDP Classifier version 2.2 against the *16S rRNA* database (Silva v138) and fungal ITS database (Unite v8.0) using a confidence threshold of 0.7. In addition, all OTUs of *16S rRNA* assigned to the chloroplast were removed for subsequent analysis.

α-diversity and β-diversity were calculated using QIIME2 with default parameters and reads rarefied to 27,222 sequencing reads per sample. A Wilcoxon rank-sum test and weighted UniFrac distances were used to test for significant differences in the α-diversity and β-diversity, respectively, between groups. Boxplots, Venn, diagrams heatmaps and columnar stacking diagrams were generated using an online tool: https://cloud.majorbio.com/page/tools/. Principal coordinate analysis (PCoA) was performed, and the results were visualized using the WGCNA, stat, and ggplot2 packages in R. Canonical correspondence analysis (CCA) was performed using the Bray‒Curtis dissimilarity matrix. The networks were generated by the R package ggClusterNet for cooccurrence and interactions. Firstly, data at the genus level were processed, and only genera with a relative abundance sum higher than 0.005 and present in five or more samples were retained. Then, Spearman’s correlation coefficient was used to evaluate the correlation of abundance, and data were kept when r >  = 0.7 and *p* < 0.05. Finally, model_Gephi.2 was selected to calculate the network attribute values and generate network diagrams.

### Supplementary Information

**Additional file 1.**

## Data Availability

The raw sequencing data can be found at the National Centre for Biotechnology Information (NCBI) Sequence Read Archive (SRA) with an accession number PRJNA1003964 (https://www.ncbi.nlm.nih.gov/sra/PRJNA1003964).
