# Supplementary material for: Effects of plant-derived protein and rapeseed oil on growth performance and gut microbiomes in rainbow trout
Source: BMC Microbiol. 2023 Sep 13;23:255. doi: 10.1186/s12866-023-02998-4 (PMC10498547; doi:10.1186/s12866-023-02998-4)
Supplement: Supplementary file 1 — Additional file 1. [file 12866_2023_2998_MOESM1_ESM.docx]

**SUPPLEMENTARY TABLES and figure**

**Supplementary Table 1:** Feed formulations for rainbow trout (g/100 g feed) of commercial-like diet (FOM), full plant protein diet (FMR) and full rapeseed oil diet (FOR).

| Diets | FOM | FMR | FOR |
| --- | --- | --- | --- |
| Ingredients |  |  |  |
| Brown fish meal | 60 | 0 | 60 |
| Soybean meal | 0 | 22 | 0 |
| Soy protein concentrate | 0 | 10 | 0 |
| Corn gluten meal | 0 | 9 | 0 |
| Wheat gluten | 0 | 8 | 0 |
| Cottonseed protein powder | 0 | 10 | 0 |
| Wheat meal | 13.5 | 13.5 | 13.5 |
| Rice bran | 5.12 | 0 | 5.12 |
| Fish oil | 18.6 | 22.52 | 0 |
| Rapeseed oil | 0 | 0 | 18.6 |
| Vitamin-mineral premix | 1 | 1 | 1 |
| Ca (H_2_PO_4_)_2_ | 0.8 | 0.8 | 0.8 |
| Choline chloride | 0.3 | 0.3 | 0.3 |
| Calcium propionate | 0.1 | 0.1 | 0.1 |
| Ethoxyquin | 0.05 | 0.05 | 0.05 |
| Betaine | 0.5 | 0.5 | 0.5 |
| Astaxanthin | 0.03 | 0.03 | 0.03 |
| Lysine HCL | 0 | 1.5 | 0 |
| Methione | 0 | 0.7 | 0 |

**Supplementary Table 2:** Dry matter and Crude protein and lipid percentage of each feed formulations for rainbow trout (% of dry matter) of commercial-like diet (FOM), full plant protein diet (FMR) and full rapeseed oil diet (FOR), and mean body weight and body Length after 84 days of feeding.

| Groups | Dry matter (%) | Crude protein  (% dry matter) | Crude lipid  (% dry matter） | body weight(g)  mean±SD | body length(cm)  mean±SD |
| --- | --- | --- | --- | --- | --- |
| FOM | 94.4 | 46.2 | 23 | 793.33±73.18 | 35.83±1.25 |
| FMR | 94.6 | 45.6 | 23.2 | 831.66±124.82 | 34.66±2.62 |
| FOR | 94.8 | 45.8 | 23.2 | 758.33±46.7 | 33.83±1.25 |

**Supplementary Table 3:** Summary of the sequencing pre-processing. The number and percentage of reads that passed the quality filter, chimera checking, and the paired-end merged reads are shown.

| **Group** | **Sample** | **Input** | **Organelle_num** | **Out_target_num** | **Chimeras_num** | **Filtered_num** |
| --- | --- | --- | --- | --- | --- | --- |
| FOM | A1 | 42691 | 248 | 5 | 4341 | 38097 |
|  | A2 | 46777 | 280 | 12 | 5047 | 41438 |
|  | A3 | 44995 | 319 | 11 | 3739 | 40926 |
|  | A4 | 42790 | 404 | 11 | 3890 | 38485 |
|  | A5 | 32184 | 271 | 13 | 2901 | 28999 |
|  | A6 | 33693 | 181 | 3 | 6773 | 26736 |
| FMR | G1 | 54132 | 28081 | 2 | 848 | 25201 |
|  | G2 | 59527 | 25444 | 8 | 863 | 33212 |
|  | G3 | 49081 | 17289 | 4 | 815 | 30973 |
|  | G4 | 57387 | 19178 | 6 | 983 | 37220 |
|  | G5 | 55702 | 46217 | 1 | 378 | 9106 |
|  | G6 | 47170 | 13753 | 2 | 887 | 32528 |
| FOR | K1 | 43694 | 322 | 2 | 784 | 42586 |
|  | K2 | 36850 | 3216 | 17 | 1777 | 31840 |
|  | K3 | 36019 | 2479 | 3 | 708 | 32829 |
|  | K4 | 46798 | 12297 | 2 | 685 | 33814 |
|  | K5 | 47913 | 6064 | 13 | 1230 | 40606 |
|  | K6 | 52161 | 11044 | 6 | 788 | 40323 |

**Supplementary Table 4**: Summary of the sequencing pre-processing. The number and percentage of reads that passed the quality filter, chimera checking, and the paired-end merged reads are shown.

| **network features** | **bacteria** | | | **fungi** | | |
| --- | --- | --- | --- | --- | --- | --- |
| groups | FOM | FMR | FOR | FOM | FMR | FOR |
| modularity_class | 3 | 6 | 7 | 3 | 6 | 6 |
| total nodes | 100 | 95 | 99 | 100 | 90 | 99 |
| total edges | 1329 | 600 | 623 | 1078 | 448 | 792 |
| positive edges | 1066 | 499 | 531 | 756 | 258 | 699 |
| negative edges | 263 | 101 | 92 | 322 | 190 | 93 |
| average degree | 26.58 | 12.632 | 12.586 | 21.56 | 9.956 | 16 |
| average weighted degree | 22.144 | 12.35 | 10.265 | 17.657 | 8.192 | 13.248 |
| network diameter | 4 | 5 | 5 | 4 | 5 | 5 |
| average path length | 2.049 | 2.482 | 2.478 | 2.146 | 2.619 | 2.415 |
| density | 0.268 | 0.134 | 0.128 | 0.218 | 0.112 | 0.163 |
| average clustering coefficient | 0.648 | 0.538 | 0.527 | 0.618 | 0.52 | 0.636 |
| total triangles | 26721 | 4617 | 4695 | 15444 | 2373 | 9606 |

**Supplementary**
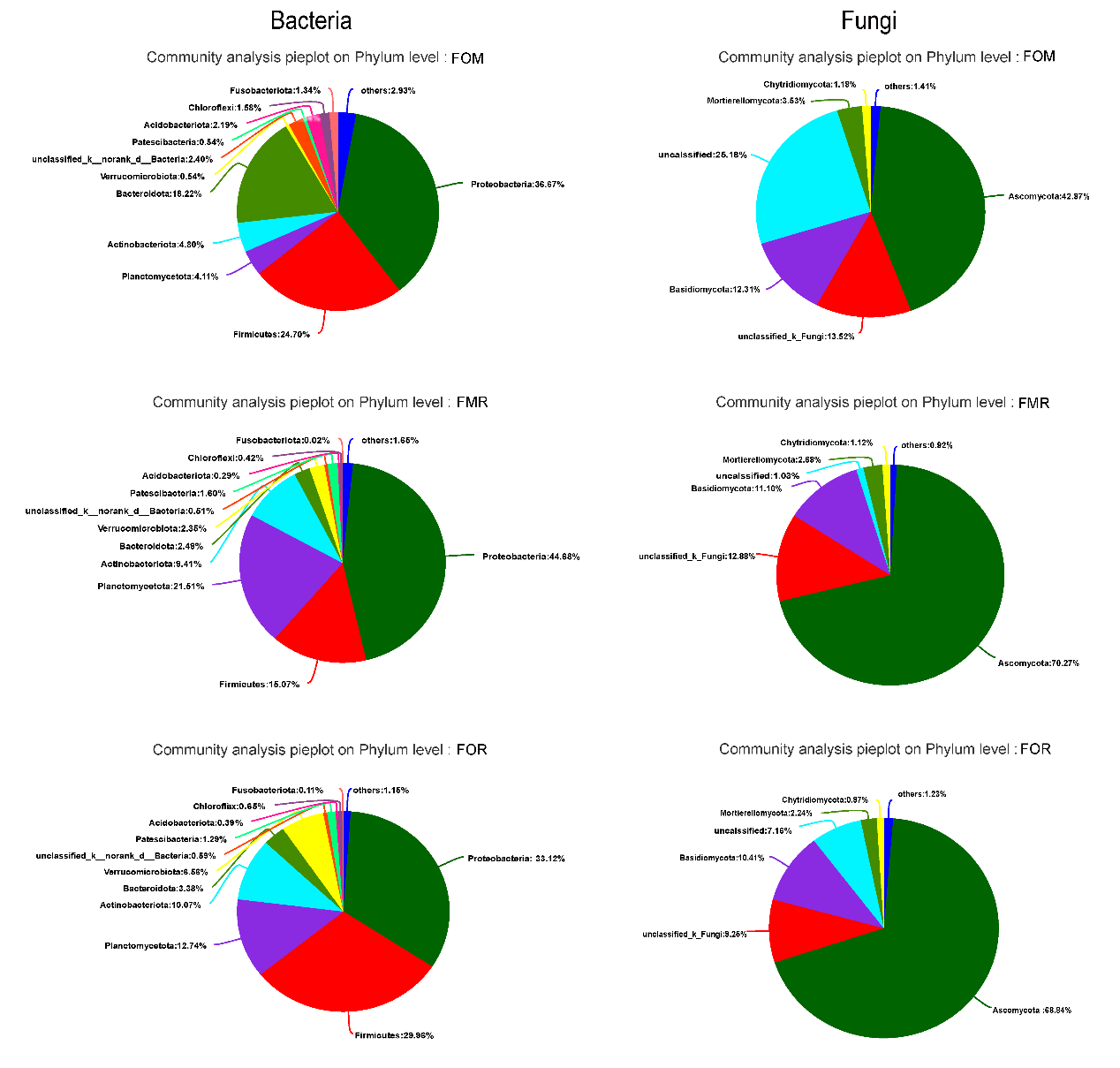
**Fig.1** Pie chart of the relative abundance of bacterial and fungal phyla (top11) in the gut microbiota in rainbow trout fed three different diets (FOM, FMR and FOR) for three months (n = 6 fish/diet); FOM: commercial diet including fish meal and fish oil, FMR: fish meal replaced with plant proteins, FOR: fish oil replaced with rapeseed oil.
